# Macrophages and vimentin in tissues adjacent to megaprostheses and mesh in reconstructive surgeries

**DOI:** 10.1080/19420889.2022.2101193

**Published:** 2022-08-05

**Authors:** Kunihiro Asanuma, Tomoki Nakamura, Takahiro Iino, Tomohito Hagi, Akihiro Sudo

**Affiliations:** Department of Orthopedic Surgery, Mie University School of Medicine, Tsu City, Japan

**Keywords:** CD68, vimentin, macrophage, megaprosthesis, mesh, soft tissue integration, reconstruction

## Abstract

In reconstructive surgery using artificial materials after wide resection, soft tissues are usually adjacent to metal surfaces or mesh. The purpose of this study was to provide histological evaluation of the soft tissues adjacent to the metal surfaces of megaprostheses and mesh. Tissues from revision surgery of megaprosthesis and from wide resection after recurrent thoracic wall sarcoma were used. Histological analysis was evaluated by hematoxylin/eosin (HE) and Masson’s trichrome staining, and by immunohistochemical staining for markers including cluster of differentiation 68 (CD68), vimentin, collagen type and S100A4. Soft tissue adherence to the smooth metal surface of Ti alloy was not observed. On the surface of capsule, CD68- and vimentin-positive cells formed a thin layer. In contrast, soft tissue adherence to a rough-surface cobalt chrome alloy was observed. Capsule was not apparent for this tissue, in which CD68- and vimentin-positive cells were aggregated randomly. In the resected tissues of recurrent chest wall sarcoma, muscles showed connections to connective soft tissues but did not invade to the inside of the mesh. Around the polypropylene mesh, large numbers of CD68- and vimentin-positive cells were seen. On the ePTFE, small numbers of CD68-positive cells were observed, while a larger number of the cells were vimentin positive. High accumulation of S100A4-positive cells was observed at the metal surface and polypropylene surface. Cells were strongly positive for CD68 and vimentin in tissues adjacent to metal and mesh surfaces. Macrophages and vimentin may play important roles in the foreign body reaction to metal and mesh, and so may contribute to encapsulation and fibrosis.

## Introduction

Bone and soft tissue sarcoma resection with a wide margin constitute some of the most successful cancer treatments supported by reliable evidence [1,2]. Wide resection sometimes is needed to resect expanses of soft tissues, bones, and chest or abdominal walls. Reconstruction is an essential procedure to retain patient motility and the function of the thoracic and abdominal cavities [3,4].

Endoprosthesis is a common device for reconstruction after bone resection for bone tumors [5]. This strategy provides a significant advantage for maintaining patient motility and quality of life. In contrast to the use of prostheses for knee or hip joints damaged by osteoarthritis, megaprosthetic replacement requires detachment of many muscles from bones; however, such detachment typically leads to functional deterioration [6]. The bone-implant junction is an extremely important site; failure at this junction leads to aseptic loosening, with an occurrence rate of 2–29% [7–11]. To reduce aseptic loosening, surface modification has been adopted to enhance conjugation between bone tissue and metal in cementless implants [12]. Furthermore, to retain high performance of extremity function, muscle power transmission to extremities is especially important. Notably, knee extension and hip abduction are major issues, and various techniques for conjugation of muscle, tendon, or bone tissue to prostheses have been considered. Some megaprostheses address this challenge by providing a porous modification at the anterior end of the proximal tibia or trochanteric major.

To recover function, anatomical reattachment of detached bone, muscles, or tendons to metal surfaces is the ideal process and should be considered. Depending on the available residual bone or loss of soft tissues, metal surfaces are connected by bone, tendon, muscle, or artificial materials. Reconstruction of knee extension is the most important theme in megaprosthetic replacement of the proximal tibia. A variety of reconstruction methods using artificial ligament, mesh, composite allograft, etc., have been reported [13–17]. In all of these procedures, the gastrocnemius flap technique is considered the “gold standard” of reconstruction methods for knee extension [18]. Additionally, for functional preservation after megaprosthetic replacement of the proximal femur, reconstruction of the gluteus medius muscle is encourage, given that failure of the hip abduction may lead to a Trendelenberg gait [19]. Groundland et al. compared the functional results of a soft tissue repair or reattachment of the bony greater trochanter to a prosthesis. Contrary to expectations, the incidence rate of a Trendelenberg gait was lower with the soft tissue repair than with reattachment of the bony greater trochanter [19]. Assuming that bone integration to the metal surface is occurring, surface modification of the prosthesis appears to provide a low rate of aseptic loosening of the stem [20]. However, surface modification of the proximal tibia component has not been used as a mainstream reconstruction method for knee extension [18]. Furthermore, the proximal femur component will not serve for hip abduction by bony greater trochanter conjugation to metal surfaces [19]. Transmission of muscle power to the prosthesis via soft tissue integration to metal is an alternative strategy that deserves further investigation.

Mesh, which typically is constructed using polypropylene (e.g., Bard^TM^ Mesh), polyester (e.g., Parietex^TM^), or similar materials, often is used for reconstructive surgery. Mesh is readily moldable and commonly is used for chest wall and abdominal wall reconstruction to maintain the anatomical cavity despite the occurrence of wall defects following wide resection. To inhibit organ adhesion, coating of the mesh (on the organ side) with expanded polytetrafluorethylene (ePTFE) has been used; one such composite material is Bard^TM^ Composix^TM^ Mesh. Moreover, mesh sometimes is applied for reconstruction after wide resection of bone and soft tissue malignant tumors in extremities. In one such strategy, polypropylene mesh is wrapped around the prosthesis, and the cut ends of muscles are sewn onto the mesh to create muscle insertions [13,14]. For instance, Trevira mesh is supplied as a knitted polyethylene terephthalate tube, and is provided as part of the Modular Tumor and Revision System (Mutars®, Implantcast Corp, Buxtehude, Germany). This mesh consists of a porous structure (porous structure, 200 µm) with a tensile strength of 4000 N, and has been used for muscle attachment [21]. Some polyester materials have been developed to bear high tensile loads. LARS® artificial ligaments consist of 90 non-woven longitudinal polyester fibers, and are able to tolerate up to 4000 N [15]. Leeds-Keio (L-K) is a polyester ligament with a maximum tensile strength of about 2200 N [22]. These materials have been used as replacements for the patellar tendon, with the choice depending on the tensile strength that is needed [15,16].

Given these precedents for reconstructive surgery using artificial materials after wide resection, soft tissues usually are adjacent to metal surfaces or mesh. However, soft tissue reaction to reconstruction materials generally (to our knowledge) has not been the focus of previous studies and has been reported only rarely. Improvements of soft tissue integration to artificial materials such as metal, mesh, or novel materials would require further characterization, for which histological analysis would be the first step. Therefore, the purpose of this study was to provide histological evaluation of the soft tissues adjacent to metal surfaces and polypropylene mesh with ePTFE, employing tissues from revision surgery using megaprostheses following wide resection after recurrent thoracic wall sarcoma.

## Materials and methods

This study was approved by the Ethics Committee of the Mie University Graduate School of Medicine (Approval No. 1310). All procedures performed in studies involving human participants were in accordance with the ethical standards of the Ethics Committee of Mie University and with the Helsinki declaration of 1975. Written, informed consent was obtained from each patient. For patients below the age 19 y, informed consent was obtained from their parents or legal guardian.

### Patient 1

**Figure 1.**
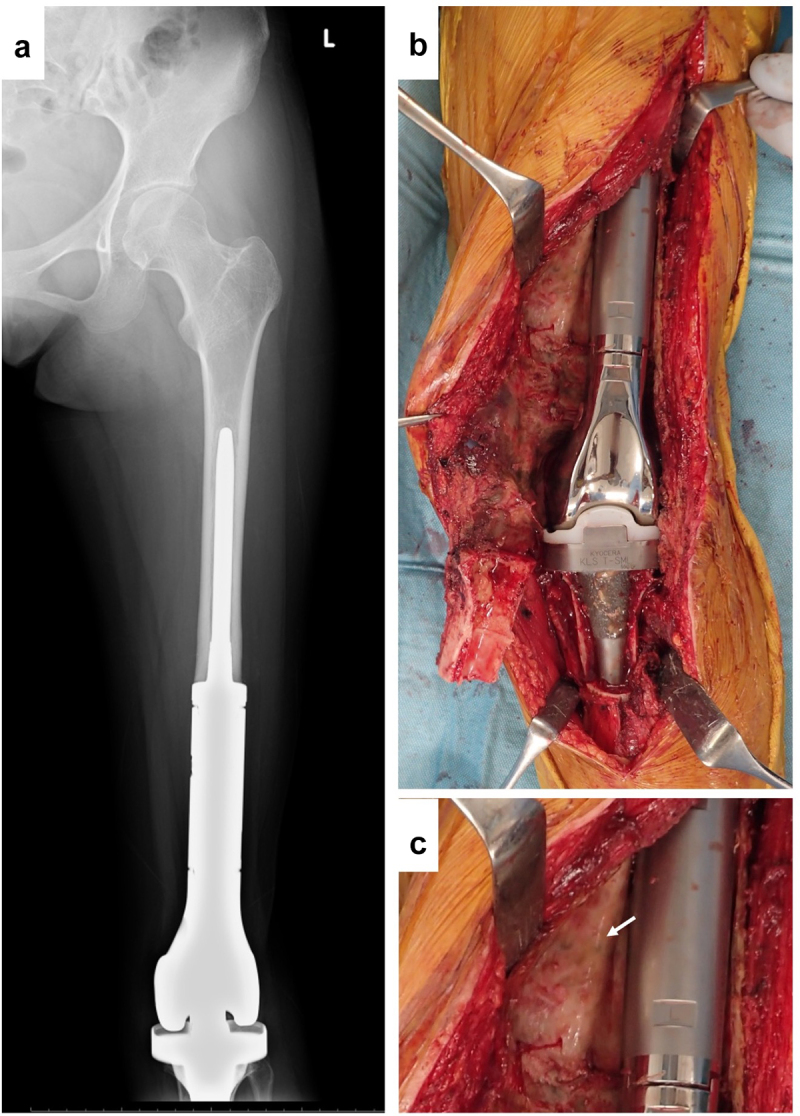
Revision by KMLS (Kyocera Modular Limb Salvage System). (a) X-ray. (b) Intraoperative picture. (c) Soft tissue adjacent to metal surface.

A 13-y-old woman received right-knee megaprosthesis replacement (KMLS: Kyocera Modular Limb Salvage System, Kyocera, Kyoto, Japan) after extraarticular wide resection due to osteosarcoma of the distal femur (Figure 1a). After 6 y, the patient developed compartment syndrome of her left leg. We diagnosed this compartment syndrome as being caused by popliteal artery stenosis, reflecting compression by the square edge of the tibial component. After the compartment syndrome was cured, the patient received revision surgery of the prosthesis and popliteal artery. After the surface of the prosthesis was reached, the connective tissues were detached carefully. The resected tissues then were submitted for histological examination.

### Patient 2

**Figure 2.**
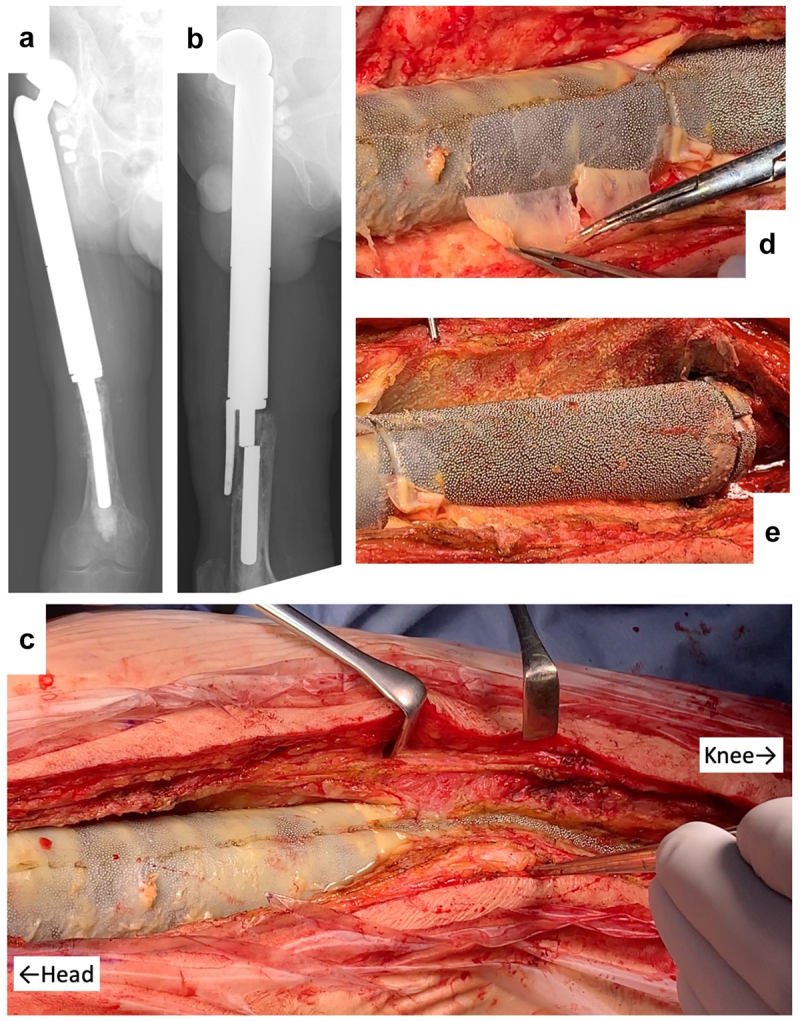
Revision by HMRS (Howmedica Modular Resection System). (a) Apical view of X-ray showing stem fracture. (b) lateral view of X-ray showing stem fracture. (c) Intraoperative picture. (d) Appearance following peeling away of thin, soft tissue. (e) Appearance following detachment of connective tissues.

A 38-y-old man received knee megaprosthesis replacement (HMRS: Howmedica Modular Resection System, Stryker, Howmedica) due to recurrence of Ewing sarcoma of the proximal femur. After 10 y, the patient underwent a revision surgery in response to infection at the HMRS. After another 10 y, the stem of the HMRS broke, necessitating further revision (Figure 2a). After the surface of the prosthesis was reached in the third surgery, the connective tissues were detached carefully from the prosthesis surface and partially harvested. These resected tissues then were submitted for histological examination.

### Patient 3

**Figure 3.**
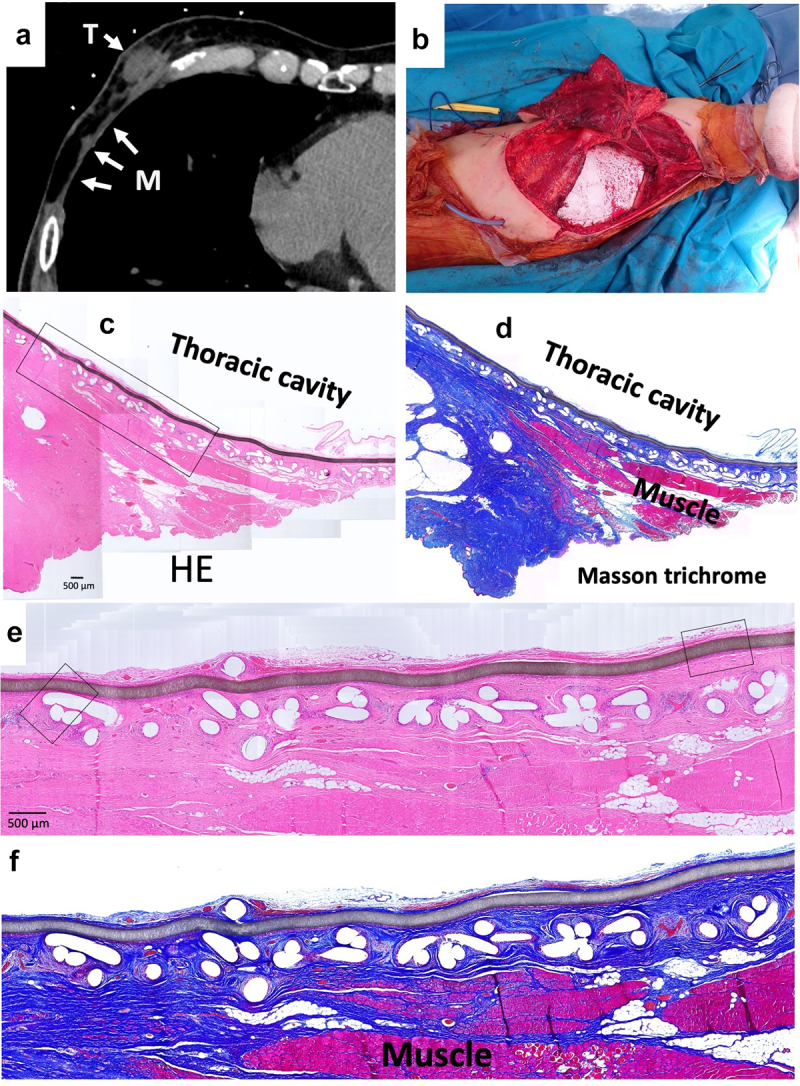
Wide resection of re-recurrent chest wall MPNST (malignant peripheral nerve sheath tumors). (a) Computed tomography of recurrent chest wall MPNST, T indicates tumor; M indicates mesh. (b) Intraoperative picture. (c) Micrograph of hematoxylin–eosin (HE)-stained section; ×40 image. (d) Micrograph of Masson’s trichrome-stained section. Red color indicates muscle tissues. (e) Micrograph of HE-stained section; ×100 image. The brown line indicates ePTFE; the aligned white defects under the ePTFE are the polypropylene mesh. (f) Micrograph of Masson’s trichrome-stained section.

A 70-y-old man suffered malignant peripheral nerve sheath tumors (MPNST) of the chest wall. When the MPNST recurred 3 y after first wide resection, Bard^TM^ Composix^TM^ Mesh was used for the chest wall reconstruction. One year later, a further recurrence was observed, necessitating another wide resection (Figure 3a, b). These resected tissues then were submitted for histological examination.

### Histological analysis

The tissues were fixed with 10% neutral buffered formalin and embedded in paraffin according to conventional methods, and then sectioned as 4-µm thicknesses using a microtome.

The sections were stained with Masson’s trichrome using Muto Pure Chemicals Co., Ltd., (Tokyo, Japan) reagents as follows. The sections were deparaffinized and incubated with the first mordant for 30 min, followed by incubation in Mayer’s hematoxylin solution for 3 min. After washing in water, the sections were incubated with 0.75% Orange G solution for 1 min. The tissue sections then were submerged in 1% acetic acid and incubated with Masson’s stain solution B for 20 min. The sections again were submerged in 1% acetic acid, incubated with 2.5% phosphotungstic acid for 10 min, submerged yet again in 1% acetic acid solution, and incubated with aniline blue solution for 30 min. The sections then were washed in ethanol, and dipped in xylene.

For immunohistochemistry (IHC), sections were subjected to paraffin removal, and antigen retrieval was conducted by heating the sections for 10 min in 10 mM citrate buffer (pH 6.0) at 120°C using a pressure cooker (CLIPSO 4 L; Tefal, Rumilly, France). The slides then were incubated overnight at room temperature with primary antibodies specific for vimentin (monoclonal mouse anti-vimentin, diluted 1:200; Catalog M0725, Dako, Jena, Denmark), CD68 (monoclonal mouse anti-CD68, diluted 1:50; Catalog M0876, Dako), CD3 (monoclonal mouse anti-CD3, diluted 1:50; Catalog M7254, Dako), CD20 (monoclonal mouse anti-CD20cy, diluted 1:200; Catalog M0755, Dako), or collagen type I (monoclonal mouse anti-hCL(I), diluted 1:500; Catalog F56, Kyowa Pharma Chemical Co., Toyama, Japan). After washing, sections were incubated for 30 min at room temperature in methanol containing 0.3% H_2_O_2_ to eliminate endogenous peroxidase activity. After washing, the slides were incubated for 30 min at 24°C with an anti-mouse immunoglobulin that had been conjugated with horseradish peroxidase (HRP) using an immuno-enzyme polymer (Histofine® Simple Stain MAX PO, Nichirei, Tokyo, Japan). All specimens were visualized in 3,3’-diaminobenzidine tetrahydrochloride (DAB) solution containing hydrogen peroxide. After washing in water, the sections were counterstained with hematoxylin.

## Results

### Soft tissue histology around a smooth metal surface (KMLS)

**Figure 4.**
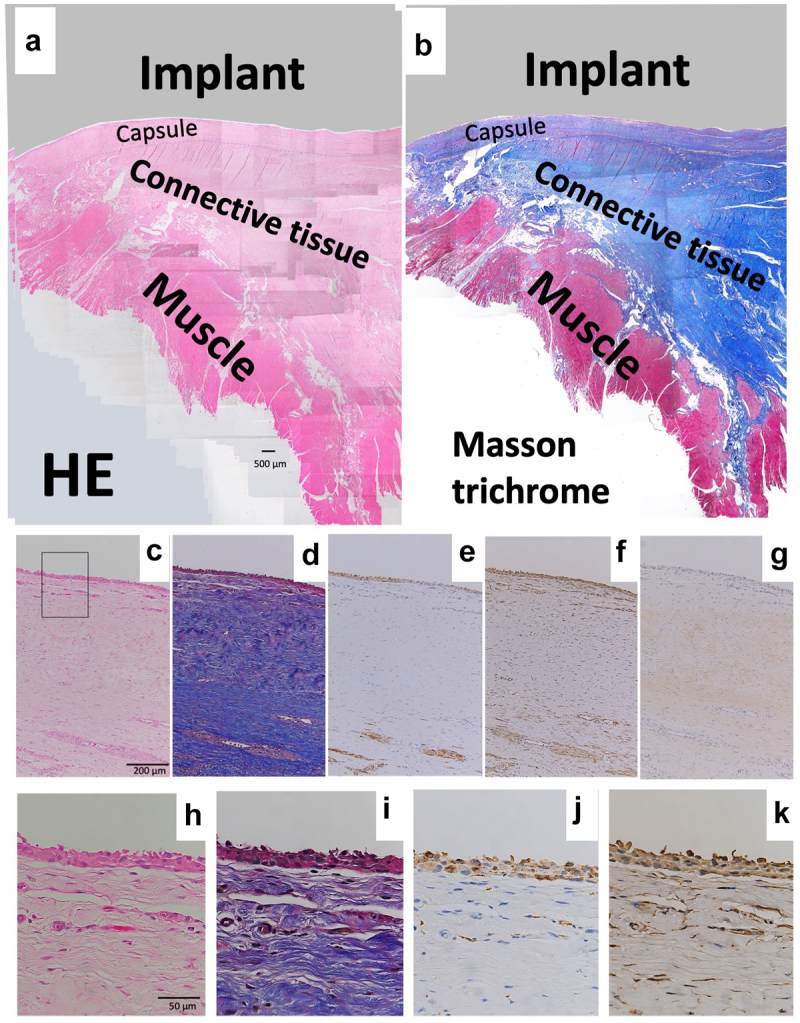
Histology of revision by KMLS. (a) Micrograph of hematoxylin–eosin (HE)-stained section; ×40 image. (b) Micrograph of Masson’s trichrome-stained section. Red color indicates muscle tissues. (c) Micrograph of hematoxylin–eosin (HE)-stained section; ×100 image. (d) Micrograph of Masson’s trichrome-stained section; ×100 image. (e) Micrograph of section with immunohistochemical (IHC) staining for CD68; ×100 image. (f) Micrograph of section with IHC staining for vimentin; ×100 image. (g) Micrograph of section with IHC staining for collagen type I; ×100 image. (h) Micrograph of HE-stained section; ×400 image. (i) Micrograph of Masson’s trichrome-stained section; ×400 image. (j) Micrograph of section with IHC staining for CD68; ×400 image. (k) Micrograph of section with IHC staining for vimentin; ×400 image.

Revision surgery for the KMLS is shown in Figure 1. The KMLS main shaft is made from Ti-6Al-4 V without coating processing. Soft tissue did not show adherence to the metal surface and was easily detached (Figure 1b). The surface of the adjacent connective tissues was circumferentially smooth (Figure 1c, arrow). These smooth tissues were used for histological examination. HE and Masson’s trichrome staining revealed the presence of capsule, connective tissues, and muscle tissues (Figure 4a, b). A thin membrane was observed on the surface of the capsule. Cells were aggregated on the thin membrane and formed a thin layer on the surface of the capsule (Figure 4c, d, h, i). IHC analysis revealed that these cells showed staining for CD68, a marker of macrophage identity (Figure 4e, j). Vimentin-positive staining was observed across a wider area than the CD68-positive area (Figure 4f, k). Slight staining for collagen type I was observed in the center of capsule tissue, though not in the cell layer (Figure 4g). No CD3 (T cell marker)- or CD20 (B cell marker)-positive cells were observed in capsule and connective tissues (data not shown). Another patient of revision surgery for the KMLS indicated same results.

### Soft tissue histology around a rough metal surface (HMRS)

Revision surgery for the HMRS is shown in Figure 2. The main shaft of the HMRS is composed of a cobalt-chrome alloy with a rough surface with gaps of approximately 0.5 mm (distance between spikes). Unlike the situation with the KMLS, soft tissues had adhered to the rough surface of the HMRS. At the head side of the prosthesis, soft connective tissues were easily detached, but the metal surface was covered by hard fibrous membrane (Figure 2c). This membrane adhered to the metal surface and did not readily peel away from the metal, such that tiny membrane fragments of tissue remained between the metal processes (Figure 2d). On the knee side of the prosthesis, soft connective tissues adhered to the metal surface and were readily peeled away from the metal (Figure 2e). These tissues were employed for histological examination.

**Figure 5.**
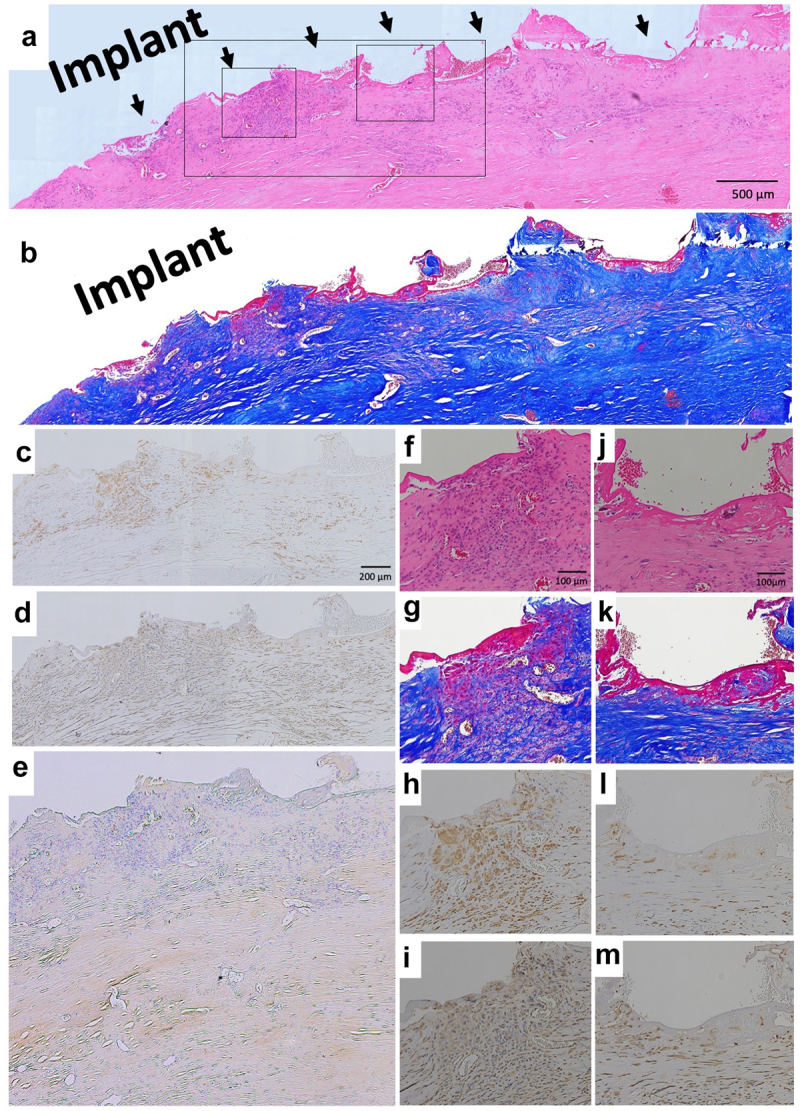
Histology of revision by HMRS. (a) Micrograph of hematoxylin–eosin (HE)-stained section; ×40 image. Arrow indicates metal process of HMRS, (b) Micrograph of Masson’s trichrome-stained section. (c) Micrograph of section with immunohistochemical (IHC) staining for CD68; ×100 image. (d) Micrograph of section with IHC staining for vimentin; ×100 image. (e) Micrograph of section with IHC staining for collagen type I; ×100 image. (f) Micrograph of HE-stained section; ×200 image. (g) Micrograph of Masson’s trichrome-stained section; ×200 image. (h) Micrograph of section with IHC staining for CD68; ×200 image. (i) Micrograph of section with IHC staining for vimentin; ×200 image. (j) Micrograph of HE-stained section; ×200 image. (k) Micrograph of Masson’s trichrome-stained section; ×200 image. (l) Micrograph of section with IHC staining for CD68; ×200 image. (m) Micrograph of section with IHC staining for vimentin; ×200 image.

HE and Masson’s trichrome staining showed the presence of a rough tissue surface along the rough metal surface. This rough tissue surface (arrows) conformed to the metal processes; upon peeling away from the metal, the tissue shape slumped (Figure 5a, arrow). Capsule was not apparent. With Masson’s trichrome staining, a thin red membrane was observed at the surface of connective tissues and was attached to the metal surface (Figure 5b, g, k). Cells showed aggregation at the second arrow from the left, but were not aggregated at the fourth to sixth arrows (Figure 5a). Most of these cells were positive for CD68 (Figure 5c, h, l) and vimentin (Figure 5d, i, m). Positive staining for collagen type I was observed in the deep layer (Figure 5e). No CD3- or CD20-positive cells were observed (data not shown).

### Soft tissue histology around ePTFE (expanded polytetrafluorethylene) and polypropylene surface

**Figure 6.**
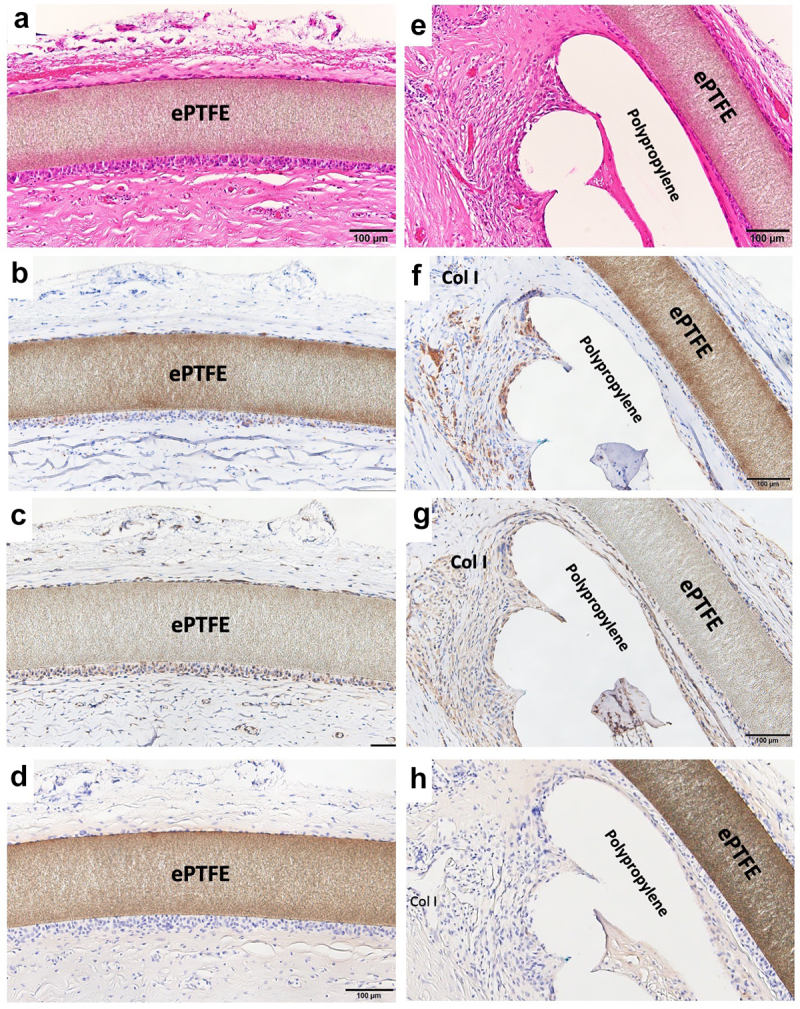
Histology of wide resection with mesh. (a) Micrograph of hematoxylin–eosin (HE) -stained section around ePTFE (expanded polytetrafluorethylene); ×200 image. (b) Micrograph of section with immunohistochemical (IHC) staining for CD68 around ePTFE; ×200 image. (c) Micrograph of section with IHC staining for vimentin around ePTFE; ×200 image. (d) Micrograph of section with IHC staining for collagen type I around ePTFE; ×200 image.

Resected tissues with Bard^TM^ Composix^TM^ Mesh without MPNST tumor are shown in Figure 3. The brown line corresponds to the ePTFE; the aligned white defects under the ePTFE correspond to polypropylene mesh (Figure 3c, e). On the thoracic cavity side, the ePTFE was covered by loose, soft tissues (Figure 3e). The polypropylene mesh side of the implant showed integrated connective tissues (Figure 3e, f). Muscles were attached to the connective soft tissues but had not invaded into the mesh (Figure 3f). On the thoracic cavity side of the ePTFE, a minority of cells showed staining for CD68 (Figure 6a). More positive staining for vimentin was observed compared to that for CD68 (Figure 6b). Only slight staining for collagen type I was seen on the ePTFE-associated tissues. No staining for CD3 or CD20 was seen in this area (data not shown). In contrast, multiple CD68-positive cells were seen in the tissues adjacent to the polypropylene mesh (Figure 6f). However, as seen for the ePTFE area, staining for vimentin was stronger than that seen for CD68 (Figure 6g). Slight staining for collagen type I was seen around the polypropylene mesh. No staining for CD3 or CD20 was not seen in this area (data not shown).

### S100A4 immunohistochemical analysis

**Figure 7.**
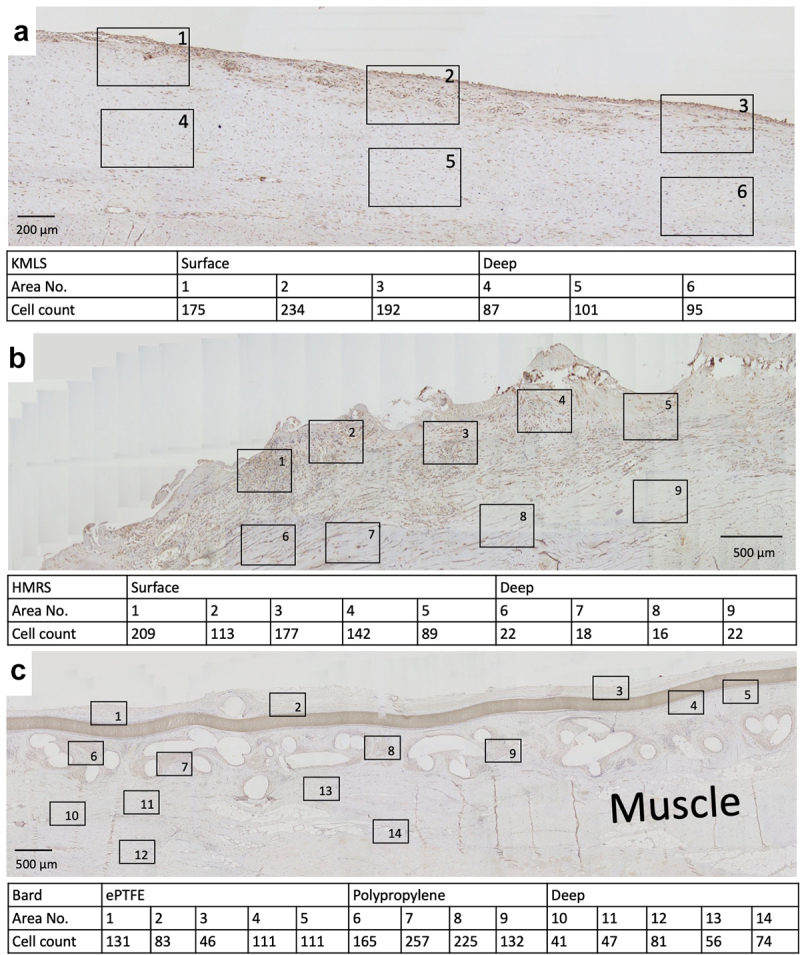
Immunohistochemical staining for S100A4. (a) Micrograph of tissue from revision by KMLS. The square indicates the counted HPF. The numbers of S100A4-positive cells are shown. (b) Micrograph of tissue from revision by HMRS. The numbers of S100A4-positive cells are shown. (c) Micrograph of tissue from wide resection with mesh. The numbers of S100A4-positive cells are shown.

**Figure 8.**
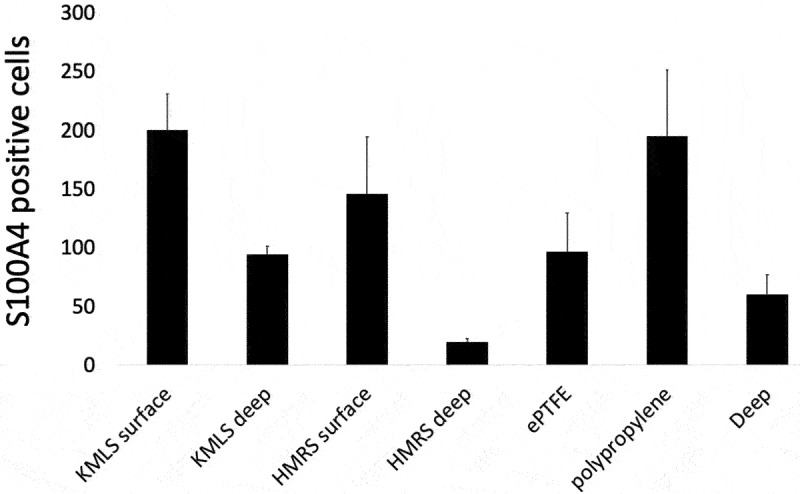
Assessment of S100A4-positive cell accumulation. Averages at the surface of KMLS, surface of HMRS, surface of ePTFE, surface of polypropylene, and their deep areas are shown.

The numbers of S100A4 (fibroblast-specific protein-1)-positive cells per high-power field (HPF) at the area adjacent to the metal surface, ePTFE, and polypropylene and their deep areas were counted. At the area adjacent to the KMLS, there was a uniform distribution of S100A4-positive cells, with an average of 200.3 cells/HPF (Figure 7a, 1–3), and an average of 94.3 cells/HPF was present at the deep area (Figure 7a, 4–6). At the area adjacent to the HMRS, accumulation of S100A4-positive cells was located roughly along the metal processes. The average number of cells at the area adjacent to the HMRS was 146 cells/HPF (Figure 7b, 1–5), whereas it was 19.5 cells/HPF at the deep area (Figure 7b, 6–9). S100A4-positive cell accumulation in tissues around ePTFE and polypropylene is shown in Figure 7c. The average at the area adjacent to ePTFE was 96.4 cells/HPF (Figure 7c, 1–5), at the area adjacent to polypropylene it was 194.8 cells/HPF (Figure 7c, 6–9), and at the deep area it was 59.8 cells/HPF (Figure 7c, 10–14). The graph of the averages is shown in Figure 8. S100A4-positive cell accumulation was roughly the same at the KMLS surface, HMRS surface, and polypropylene, whereas the accumulation at ePTFE was slightly less.

## Discussion

In this study, we performed histological analysis of the soft tissues adjacent to megaprostheses and mesh using surgical tissues recovered from clinical cases. Biomaterial implantation induces foreign body response, and the degree of this response differs depending on the properties of the implanted material. The biological process of the foreign body response follows a defined course that includes (sequentially) provisional matrix formation, acute inflammation, chronic inflammation, and fibrous capsule formation [23–25]. Macrophages contribute to the inflammatory response by releasing pro-inflammatory cytokines during the acute inflammatory phase [26–28]. During the chronic inflammation phase, interactions between the macrophages and fibroblasts drive encapsulation over a 3- to 4-week interval [28,29]. Macrophages play an important role in the foreign body response, notably during the stages extending from inflammation to encapsulation. In the present study, we conducted histological evaluation of tissues that had been adjacent to metal surface for 6 and 10 y after implantation and adjacent to mesh for 1 y after implantation.

Titanium and titanium alloys are thought to be bioinert for bone connection. Bone tissues have been shown to regenerate along titanium surfaces [30]. To enhance the connection between bone and Ti, various surface modification techniques have been used, including grit blast, plasma spray, fiber mesh, beads, three-dimensional porous surfaces, etc. [31,32]. Further investigations are continuing worldwide in an effort to reduce loosening and improve stability of the bone-prosthesis connection. In contrast, the soft tissue reaction to metal surfaces has not (to our knowledge) been the similar subject of experimental focus. Unlike the prostheses employed in response to osteoarthritis, megaprostheses usually touch spacious soft tissues and therefore need to address at least two aspects specific to the soft tissue environment. The first is the integration between the muscle or tendon and the metal surface to transmit muscle power. The second is the need for soft tissues to not adhere to metal surfaces where tissue sliding around the joint is required, for example on the anterior distal femur. In the present work, we evaluated tissue histology adjacent to two different prostheses. One was a smooth-surface Ti alloy, and the second was a rough-surface cobalt-chrome alloy.

In Figure 1 b and c, tissues did not show connections to the smooth Ti alloy surface. Histologically, capsule was formed around this metal surface (Figure 4a, b). This encapsulation of the prosthesis probably reflected a foreign body reaction. On the surface of the capsule, macrophages (CD68-positive cells) formed a thin layer (Figure 4h–k). In contrast, on the rough surface of the cobalt-chrome alloy, soft tissues showed adherence to the metal surface (Figure 2c–e). No capsule was apparent, though thick connective tissues were seen (Figure 5a, b). CD68-positive cells accumulated randomly along this interface (Figure 5c). Ungersböck et al. reported that the roughness of a metal surface influences the soft tissue reaction and capsule thickness, such that rough surfaces show thinner capsules [33]. This difference compared to our data probably reflects the specific surface modification of the metal implant. Additionally, in our histological examination, strong staining for vimentin was widely seen across the CD68-positive area (Figure 4f, 5d). Indeed, macrophages activated by cytokines are known to secrete vimentin into the extracellular space [34]. It has been reported that vimentin controls fibroblast proliferation and collagen accumulation, as demonstrated using a vimentin-deficient mouse model [35,36]. Vimentin has the potential to induce fibrosis by inducing mesenchymal cells to differentiate into myofibroblasts [37]. In general, macrophages are known to play an important role in the control of fibroblasts and inflammatory cells, and have been shown to regulate fibrogenesis by producing growth factors and cytokines [38]. We speculate that metal surfaces induce macrophage reaction, leading to fibrosis via vimentin secretion. S100A4 (fibroblast-specific protein-1) is well known as a general marker of fibroblasts, and the number of S100A4-positive cells has usually been used for assessment of tissue fibrosis [39]. The number of S100A4-positive cells per HPF was evaluated in the area indicated in Figure 7; the average at the HMRS surface was slightly less than the average at the KMLS surface, with no significant difference (Figure 8). This slight difference may contribute to capsule thickness. The specific surface modification of the metal implant may affect fibroblast production. For next-generation prostheses, material engineering is expected to have progressed sufficiently to nanoscale technology to permit the control of cell behavior, such that nano-level surface modifications may provide control of cell reactions. Ariganello et al. reported that nanocavitated surfaces show decreased cytokine production from macrophages [40]. Wang et al. reported that nanotubes enhance fibroblast proliferation and extracellular matrix (ECM) production [41]. In separate work, implants with TiO nanotubes resulted in decreased numbers of CD68-positive cells and thinner capsules [42]. Thus, it appears that nanotechnology has the potential to control cell reactions adjacent to metal surfaces.

Other studies have examined the mechanical strength of soft tissue integration onto metal surfaces in vivo. In 1982, Bobyn et al. measured peeling strength using implants in adult mongrel dogs [43]. These researchers used cobalt-based alloy plates that were generated with three different pore sizes: fine (5–20 µm), medium (20–50 µm), and coarse (50–200 µm). The plates were Implanted into subcutaneous tissue and harvested 16 weeks later. Based on mechanical analysis, the peel strength of tissue attachment was strongest with the coarse plate [43]. In 2020, Tikhilov et al. measured tensile strength of soft tissue by inserting Ti-6Al-4 V plates (20 mm × 7 mm × 3 mm, 100-µm pore size) into the latissimus dorsi muscle of 7-month-old female Chinchilla rabbits for 90 d. The strength of the soft tissue adhesion to the 100-µm-pore plates was higher than that obtained with non-porous plates [44]. This result emphasizes that surface modification can regulate soft tissue integration onto a metal surface. To date, metal surfaces with 100-µm pores appear to show the best mechanical integration of soft tissue to a metal surface. In the future, we believed further soft tissue integration to transmit muscle power may be needed for next-generation megaprostheses. We expect that nanoscale technology will improve soft tissue integration and tensile strength for integration with metal surfaces. Mesh substrates made from polypropylene, polyester, and ePTFE are major artificial materials for surgical reconstruction. For chest or abdominal wall defects, mesh or flap are the most-used methods for chest wall or abdominal wall reconstruction seeking to maintain anatomical thoracic and abdominal cavities [4,45]. Mesh sometimes also is applied for reconstruction after wide resection of bone and soft tissue malignant tumors in extremities, especially for knee extension. However, histology around mesh used for reconstruction after wide resection in human has been reported only rarely, to our knowledge. To improve mesh quality, histological analysis is important for elucidating cellular or tissue reactions to mesh.

Mesh frequently induces macrophage accumulation due to chronic foreign body reaction. The degree of this response differs based on the mesh material, number of filaments, and the presence or absence of knots [46,47]. Osanai et al. evaluated the histology of the L-K artificial polyester ligament used in surgical revision materials; the results indicated slight inflammation in the fibrous tissues [48]. In our data, macrophage (CD68-positive cells) accumulated in the tissues adjacent to polypropylene mesh (Figure 6c). Vimentin was widely and strongly positive in the same area (Figure 6d). On the surface of ePTFE, slight staining for CD68 was seen (Figure 6a); staining for vimentin was widely positive, more so than for CD68 (Figure 6b). In contrast, Wolf et al. reported that ECM coating of mesh with porcine urinary bladder matrix or porcine dermal matrix decreases macrophage accumulation around mesh in the Sprague Dawley rat [49]. This observation indicates that it may be possible to control the cell and tissue inflammatory reaction caused by mesh via mesh modification. Additionally, histology of soft tissue integration into L-K ligaments showed that muscle tissues were not integrated into L-K ligaments directly, instead connecting to the L-K ligaments via fibrous tissues. [48]. Similarly, in our data, muscle did not integrate into the mesh, instead attaching to polypropylene fibers via connective tissues. Some mesh materials have been developed to bear high tensile load of 2000–4000 N [15,21,22]. However, connective tissues inevitably intervene between muscle tissues and mesh. High tensile strength of the regenerated connective tissues is needed. We expect that the development of new materials or new surface modifications of mesh will decrease the cell reaction while enhancing the strength of integration of soft tissues into mesh.

The ideal material for prosthetic implants would exhibit rapid wound healing, minimal chronic inflammation, minimal foreign body reaction, resistance to infection, and rapid soft tissue integration [29,47]. Furthermore, given that megaprotheses often are used for reconstruction after wide resection of extremities, fibrous connective tissues around metal and mesh need to maintain high tensile strength to transmit muscle power to such prostheses.

## Conclusion

This study employed histological analysis of tissues adjacent to megaprostheses and mesh in tissues recovered from clinical cases. Staining for CD68 and vimentin was strongly positive in the tissues adjacent to metal and mesh. We infer that macrophages and vimentin may play important roles in the foreign body reaction to metal and mesh, and that these reactions contribute to encapsulation and fibrosis. It is expected that surface modifications of metal and mesh surfaces will improve cell reaction and tensile strength of regenerated soft tissues adjacent to implanted protheses.

### Limitation

The number of patients was small, and alloy was different. However, revision surgery of megaprosthesis and wide resection including mesh after recurrent thoracic wall sarcoma were extremely rare. We believed this is a chance to provide histological evaluation of the soft tissues reaction adjacent to metal surfaces and polypropylene mesh in human.
